# Differential Dopamine D1 and D3 Receptor Modulation and Expression in the Spinal Cord of Two Mouse Models of Restless Legs Syndrome

**DOI:** 10.3389/fnbeh.2018.00199

**Published:** 2018-09-04

**Authors:** Samantha Meneely, Mai-Lynne Dinkins, Miki Kassai, Shangru Lyu, Yuning Liu, Chien-Te Lin, Kori Brewer, Yuqing Li, Stefan Clemens

**Affiliations:** ^1^Department of Physiology, Brody School of Medicine, East Carolina University, Greenville, NC, United States; ^2^Department of Neurology, College of Medicine, University of Florida, Gainesville, FL, United States; ^3^East Carolina Diabetes and Obesity Institute, Brody School of Medicine, East Carolina University, Greenville, NC, United States; ^4^Department of Emergency Medicine, Brody School of Medicine, East Carolina University, Greenville, NC, United States; ^5^Wuxi Medical School, Jiangnan University, Wuxi, China

**Keywords:** RLS animal models, dopamine, D1 receptor, D3 receptor, Meis1, sensorimotor function, spinal cord

## Abstract

Restless Legs Syndrome (RLS) is often and successfully treated with dopamine receptor agonists that target the inhibitory D3 receptor subtype, however there is no clinical evidence of a D3 receptor dysfunction in RLS patients. In contrast, genome-wide association studies in RLS patients have established that a mutation of the *MEIS1* gene is associated with an increased risk in developing RLS, but the effect of *MEIS1* dysfunction on sensorimotor function remain unknown. Mouse models for a dysfunctional D3 receptor (D3KO) and Meis1 (Meis1KO) were developed independently, and each animal expresses some features associated with RLS in the clinic, but they have not been compared in their responsiveness to treatment options used in the clinic. We here confirm that D3KO and Meis1KO animals show increased locomotor activities, but that only D3KO show an increased sensory excitability to thermal stimuli. Next we compared the effects of dopaminergics and opioids in both animal models, and we assessed D1 and D3 dopamine receptor expression in the spinal cord, the gateway for sensorimotor processing. We found that Meis1KO share most of the tested behavioral properties with their wild type (WT) controls, including the modulation of the thermal pain withdrawal reflex by morphine, L-DOPA and D3 receptor (D3R) agonists and antagonists. However, Meis1KO and D3KO were behaviorally more similar to each other than to WT when tested with D1 receptor (D1R) agonists and antagonists. Subsequent Western blot analyses of D1R and D3R protein expression in the spinal cord revealed a significant increase in D1R but not D3R expression in Meis1KO and D3KO over WT controls. As the D3R is mostly present in the dorsal spinal cord where it has been shown to modulate sensory pathways, while activation of the D1Rs can activate motoneurons in the ventral spinal cord, we speculate that D3KO and Meis1KO represent two complementary animal models for RLS, in which the mechanisms of sensory (D3R-mediated) and motor (D1R-mediated) dysfunctions can be differentially explored.

## Introduction

### Background on RLS

Restless Legs Syndrome (RLS) is a highly prevalent (5–10% of the population, Ghorayeb and Tison, 2010; Earley et al., 2011), but also underappreciated aging-associated neurological sensorimotor disorder that severely disrupts sleep and affects quality of life. First described in 1685 (Willis, 1685) and clinically confirmed in the mid-twentieth century (Ekbom, 1944, 1945, 1960), RLS is a clinical disorder in which overlapping genetic risk factors may play a role in the emergence of the symptoms (Trenkwalder et al., 2016). Genome-wide association studies in RLS patients have established several chromosome loci associated with RLS, notably *MEIS1* and *BTBD9* (Stefansson et al., 2007; Winkelmann et al., 2007), of which a point mutation in the *MEIS1* gene has the highest odd ratios with RLS (Winkelmann, 2008; Schormair et al., 2011, 2017; Winkelmann et al., 2011). Meis1 plays a role in the early development of the nervous system (Spieler et al., 2014; Marcos et al., 2015), and is essential to specify cell fates and differentiation patterns along the proximodistal axis of the limbs (Mercader et al., 1999, 2009). Intriguingly, a dysfunction of the *MEIS1* homolog in *C. elegans* is associated with an altered projection phenotype of dopamine neurons (M. Aschner, Albert Einstein College of Medicine, personal communication), suggesting a possible interaction between *MEIS1* and dopamine (DA) function.

RLS is often and successfully treated with DA receptor agonists that target the inhibitory D2-like receptor subtype, in particular D3 (Stiasny et al., 2000; Ferri et al., 2010; Manconi et al., 2011a; Garcia-Borreguero et al., 2016), and it has been suggested that a dysfunction of the descending A11 DA system in the hypothalamus may be involved in RLS by predominantly affecting the D3R system (Clemens et al., 2006; Lanza et al., 2017). There exist three DA receptors that mediate inhibitory actions via G*i*-coupled pathways (D2, D3, and D4), and the D3R subtype has a very high affinity to DA (Robinson et al., 1994; Cote and Kuzhikandathil, 2014). Yet despite the efficacy of the D3R compounds in treating RLS symptoms, there is no clinical evidence of A11 dysfunction in patients (Earley et al., 2009). However, a dysfunction of the DA system has been linked to altered iron homeostasis or iron-deficient diet (Dowling et al., 2011; Klinker et al., 2011; Dauvilliers and Winkelmann, 2013; Earley et al., 2014).

### Animal models of RLS

Mouse models for a dysfunctional D3 receptor (D3KO) and Meis1 (Meis1KO) were developed independently (Accili et al., 1996), and each animal expresses some features associated with RLS in the clinic. Both D3KO and Meis1KO express increased locomotor activity (Accili et al., 1996; Salminen et al., 2017), but only D3KO show an increased sensory excitability both in the isolated spinal cord (Clemens and Hochman, 2004) and *in vivo* (Keeler et al., 2012). The thermal pain withdrawal reflex depends on spinal cord circuits that can be recruited experimentally to assess compromised function of the underlying neural networks, both with the spinal cord and extending into the periphery. The previously reported increased excitability of the D3KO mouse to thermal stimuli suggests a possible role of C-fiber mediated pathways that convey altered sensations from deep within the muscle tissue (Clemens et al., 2006; Keeler et al., 2012). Further, as recent data suggest that the inhibitory D3 receptor can form functional heteromeric dimers with the excitatory D1 receptor (Marcellino et al., 2008), it is conceivable that the increased excitability observed in D3KO may be the result of an increased expression of the excitatory dopamine D1 receptor (D1R) (Brewer et al., 2014).

We here compared the effects of dopaminergic treatment on spinal reflexes as a tool to assess sensorimotor function (Eccles and Lundberg, 1959; Nielsen, 2004; Barriere et al., 2005) in Meis1KO and D3KO animal models, and we assessed D1R and D3R expressions the spinal cord, the gateway for sensorimotor processing. We found that Meis1KO share most of the tested behavioral properties with their wild type (WT) controls, including the modulation of the thermal pain withdrawal reflex by morphine, L-DOPA and D3 receptor (D3R) agonists and antagonists. However, Meis1KO and D3KO also shared behavioral similarities when tested with D1 receptor (D1R) agonists and antagonists, while the matching WTs were unresponsive to these drugs. Subsequent Western blot analyses of D1R and D3R protein expression in the spinal cord revealed a significant increase in D1R expression in Meis1KO and D3KO over the WT controls, while D3R expression was not significantly different across all 3 groups. Our data show that changes in spinal D1R expression and behavioral responses to D1R compounds are similar between Meis1KO and D3KO, while the increased sensitivity at baseline is present only in D3KO. We provide a model that separates D3KO and Meis1KO into two complimentary models of RLS that represent sensory and motor dysfunctions, respectively.

## Methods

### Animals

All experimental procedures were approved by the Institutional Animal Care and Use Committees at East Carolina University and University of Florida, and were fully compliant with the National Institutes of Health guide for the care and use of Laboratory animals (NIH Publications No. 80-23). All efforts were made to minimize the number of animals used, and a total of 55 male mice (age range ~9–12 months) were tested in this study. Behavioral testing was performed on *Meis1* heterozygous knockout mice (*Meis1*+/Δ, Meis1KO, *n* = 15) and their appropriate wild-type (WT) controls (C57BL/6J, *n* = 12), dopamine D3 receptor knockout mice (D3KO; strain B6.129S4-Drd3^tm1dac^/J (stock # 002958, Jackson Laboratory, Bar Harbor, ME), *n* = 13) and their appropriate WT controls (C57BL/6J, *n* = 15) (Clemens and Hochman, 2004; Brewer et al., 2014). *Meis1 loxP mice* were from Drs. Copeland and Sadek (Kocabas et al., 2012), and Meis1+/Δ were generated by crossing *Meis1 loxP* with Emx1-cre mice (Guo et al., 2000). *Emx1* expresses in testis germ cells, so that when germ cells contain both *Emx1*-cre and *Meis1 loxP*, cre-mediated recombination occurs and leads to a deletion of the *loxP*-flanked sequence. Male mice heterozygous for both *Meis1 loxP* and *Emx1*-cre were then bred with WT female mice. The deletion of *Meis1* gene occurred during the development and *Meis1*+/Δ mice were derived. Finally, *Meis1*+/Δ mice were crossed with WT mice to generate experimental mice. Animals were housed with free access to food, water, and enrichments under a 12-h light/dark cycle at room temperature.

### Behavioral assessments–hargreaves

Behavioral testing procedures have been described in detail recently (Keeler et al., 2012; Brewer et al., 2014). Thermal withdrawal latencies (Hargreaves' method) were obtained in each cohort by using the IITC plantar analgesia meter (IITC Series 8, IITC Inc., Woodland Hills, CA). Experiments were performed between 9 am and 1 pm, to minimize the circadian variation. The week before testing started, animals were acclimated on 3–4 days to the experimental room and the Hargreave's system, by placing them individually into the Plexiglas cubicles for an average of 2 h. In week 1 of the testing period, we tested the effects of vehicle injections (0.9% NaCl, i.p., ~90–120 μl per animal). Animals were tested 5 times per session, with resting periods for each individual animal between tests of 5–10 min. Stimulation cut-off for each test was set to 30 s test duration, to prevent the possibility of a heat-induced injury. Once initiated, recording sessions for all 5 trials lasted no longer than 60–90 min for all animals tested that day. After vehicle assessments, we subsequently compared all drug effects (i.p. injections, injection volumes matching the vehicle volumes) against the data obtained after the respective vehicle injections in each animal cohort. We started the tests 1 h after vehicle or drug injections. Each drug test was separated from the next drug treatment by an at least 3-day recovery period, to minimize any potential drug interactions possibly skewing the latency measurements. After ~5–6 weeks, we again tested the responses to vehicle injections in two cohorts and found no significant differences from the values obtained at baseline (data not shown).

### Behavioral assessments–locomotor activity

Spontaneous locomotor activities were recorded for Meis1KO and their WT controls with a VersaMax Legacy open field apparatus connected to a computerized Digiscan System (Accuscan Instruments, Inc. OH), and for D3KO and their controls with a TSE LabMaster System (TSE Systems, Chesterfield, MO). Infrared sensors were used to record ambulatory activity in the X-Y plane). Counts across all these axes were summed to give total ambulatory activity. Meis1KO and their WT controls were monitored for 7 days. Only the data from the last 4 days were pooled and analyzed. For D3KO and their controls, after 2 days of acclimation, data were collected for 2 consecutive days to calculate locomotor activity.

### Compounds tested

We tested the effects of levodopa (L-DOPA, 10 mg/kg, Acros Organics, Geel, Belgium), the D3 receptor agonist, pramipexole (0.5 mg/kg, ApexBio Technology LLC, Houston, TX), the D3 receptor antagonist, SB277011-A (10 mg/kg, Abcam Cambridge, MA), the D1 agonist, SKF 38393 (10 mg/kg, Tocris, Ellisville, MO), the D1 receptor antagonist, SCH 39166 (5 mg/kg, Tocris, Ellisville, MO), and morphine (morphine sulfate salt pentahydrate, 2 mg/kg, Sigma-Aldrich St. Louis, MO). Drug concentrations were chosen based on previous publications by others and us (Acquas and Di Chiara, 1999; Williams et al., 2006; Brewer et al., 2014; Solís et al., 2015; Dinkins et al., 2017)

### Tissue harvesting and protein isolation

Mice were deeply anesthetized with isoflurane and decapitated before spinal cords were dissected out, immediately placed in RNAlater (Thermo Fisher Scientific, Waltham, MA), and stored at −20°C until use. Spinal cords were homogenized in 1 ml of RIPA buffer with protease and phosphatase inhibitors (0.12 ml/ml RIPA buffer, Sigma-Aldrich #P2714 and 0.012 ml/ml of RIPA buffer, Sigma-Aldrich #P5726 St. Louis, MO, respectively). The homogenized spinal cords were centrifuged (13,000 rpm, 4°C, 15 min), and supernatants were aliquoted and stored individually at −80°C. Following homogenization, standard protein concentrations were established with a Bradford protein assay (Quickstart Bradford reagent, Bio-Rad #500205), and plates were read on an Epoch Microplate Spectrophotometer (BioTek, Winooski, VT) at 595 nm using the Gen5.1 software package (BioTek, Winooski, VT).

### Western blot

For Western blots, 30 μg of each lumbar spinal cord protein samples were denatured using 2x Laemmli buffer containing 5% β-mercaptoethanol and 1% SDS at 95°C for 10 min and loaded onto a 12% Criterion™ TGX Stain-Free™ Protein Gel (#5678045, Bio-Rad, Hercules, CA) and run ~45 min at 200 V. The proteins were transferred to a low fluorescent PVDF membrane using Trans-Blot® Turbo™ RTA Midi LF PVDF Transfer Kit (#170-4275, Bio-Rad, Hercules, CA). Membranes were probed with the primary antibodies and with secondary antibodies (LI-COR, Lincoln, NE) by the iBind™ Flex Western Device (SLF2000, Thermo Fisher, Waltham, MA) based on sequential Lateral Flow (SLF) technology using iBind™ Flex Fluorescent Detection (FD) Solution Kit (SLF 2019, Thermo Fisher, Waltham, MA). Membranes were imaged with an Odyssey imaging system (Odyssey Clx, LI-COR, Lincoln, NE).

### Antibodies

The primary antibodies used for Western blot to detect receptor-specific protein expression were: anti-dopamine receptor D1 (Abcam 78021, 1:500 Cambridge, MA) and anti-dopamine receptor D3 (Abcam 42114, 1:1,000 Cambridge, MA). The secondary antibodies used were goat anti-rabbit 680RD (925-68071, 1:4,000, LI-COR Biosciences, Lincoln, NE) and goat anti-mouse 680RD (925-68070, 1:4,000, LI-COR Biosciences, Lincoln, NE).

### Statistical analysis

Following the experiments, behavioral data were transferred and stored in Excel format, then analyzed and plotted offline with SigmaPlot (Version 11, SPSS Science, San Jose, California). For statistical comparisons, we employed parametric or non-parametric comparisons as appropriate when comparing multiple groups (One-Way ANOVA, RM ANOVA, or ANOVA on Ranks) with appropriate *post-hoc* comparisons (Holms-Sidak, Dunn's); *t*-tests or paired *t*-tests were used for comparison between two sets of data (treatment against respective control vehicle treatment only). Significance levels were set at *p* < 0.05. For Western blot analysis, images were analyzed with ImageStudio and ImageJ (1.50i National Institutes of Health, USA) and statistical analyses were performed with SigmaPlot 11.0.

## Results

### Thermal pain withdrawal latencies under baseline conditions

We first tested and compared pain withdrawal latency responses in Meis1KO and D3KO lines under baseline conditions (after sham injection, i.p., 0.9% NaCl, 100 μl/30 g), and compared them to their respective controls. Meis1KO and their respective controls were tested as two independent cohorts. In cohort 1 the average WT latency was 10.2 ± 1.1 s, while Meis1KO latencies were 10.2 ± 0.5 s (*p* = 0.97, *n* = 7 each, power: 0.05). In cohort 2 (tested about 1 year later) the average WT latency was 6.6 ± 0.4 s, while Meis1KO latencies were 6.7 ± 0.9 s (*p* = 0.92, *n* = 6 [WT] and *n* = 7 [Meis1KO], power: 0.05). Thus despite the difference between the two Meis1KO cohorts and their WT controls, there was no effect within each cohort between WT and knockout. In contrast, individual withdrawal latencies in D3KO ranged per trial from 4.8 to 11.1 s, (average: 8.5 ± 0.24 s, *n* = 7) while the latencies of their respective WTs (WT_*D*3*KO*_) ranged from 5.9 to 13.7 s (average: 6.5 ± 0.33 s, *n* = 7). A *t*-test comparison revealed that the difference between WT_*D*3*KO*_ and D3KO was significant (*p* < 0.001, power: 0.99).

**Figure 1:**
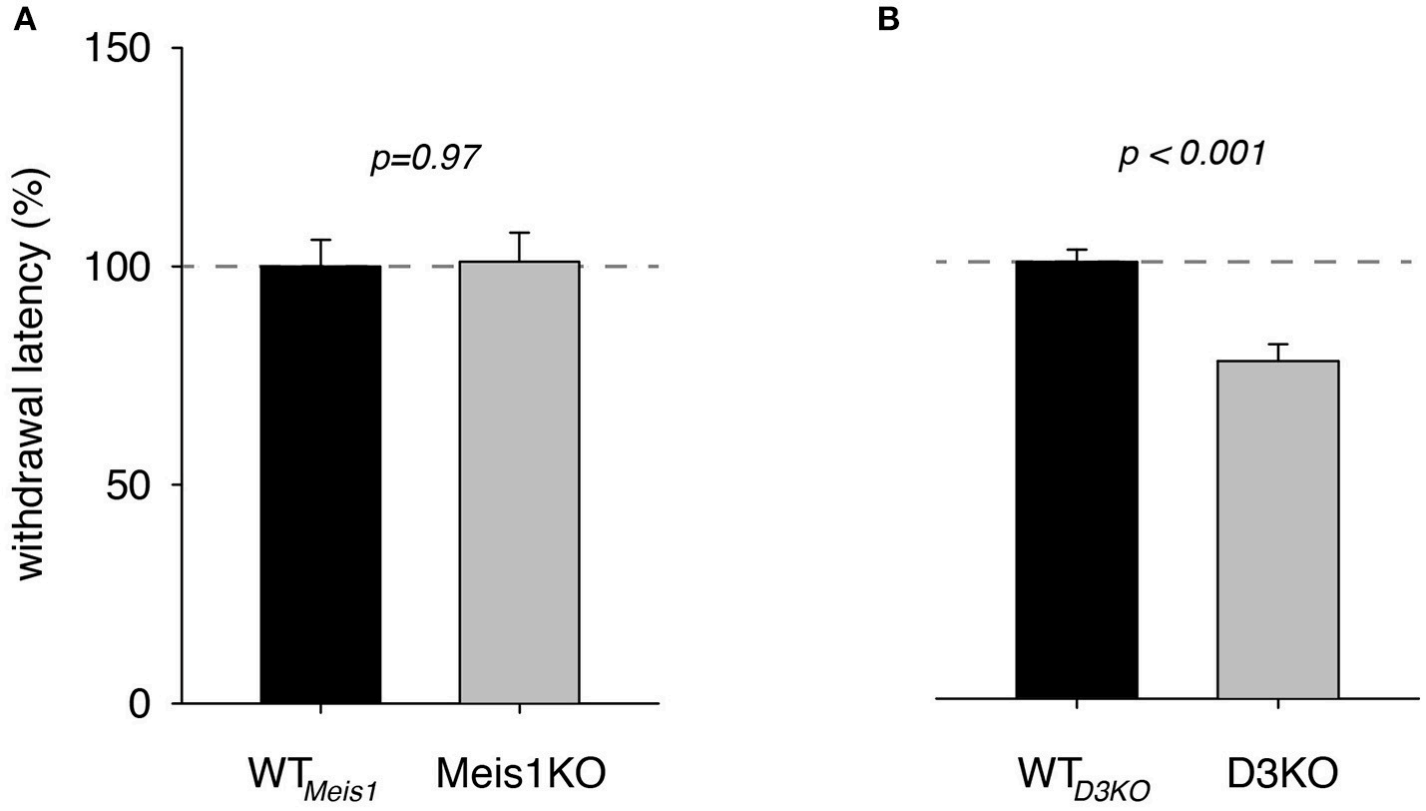
Thermal pain withdrawal latencies under vehicle (control) conditions. **(A)** Normalized representation of data from Meis1KO mice and their respective WT controls (WT_*meis*1_). WT _*meis*1_ latencies (black bar) were similar to those of Meis1KO (gray bar). **(B)** Normalized representation of data from D3KO animals and respective WT controls (WT _*D*3*KO*_). WT _*D*3*KO*_ latencies (black bar) were significantly higher than those of the D3KO (gray bar).

To illustrate the effect of the genotype alone on thermal pain withdrawal reflex latencies, we normalized the WT_*meis*1_ response to 100% and compared them with the effects in the genetically modified animals (Figure 1). We found that pain withdrawal latencies of Meis1KO and their respective controls (WT_*meis*1_) were not significantly different from each other (WT_*meis*1_: 99.9 ± 6.1% S.E. *n* = 13, Meis1KO: 101 ± 6.7 % S.E., *n* = 14, *p* = 0.97, *t*-test, power: 0.5), while those of D3KO were significantly reduced over their respective WTs (WT_*D*3*KO*_: 99.99 ± 2.8%; D3KO: 77.3 ± 3.9%, S.E., *p* < 0.001, *t*-test, both: *n* = 7, power: 0.99).

### Morphine modulation of withdrawal latencies

**Figure 2:**
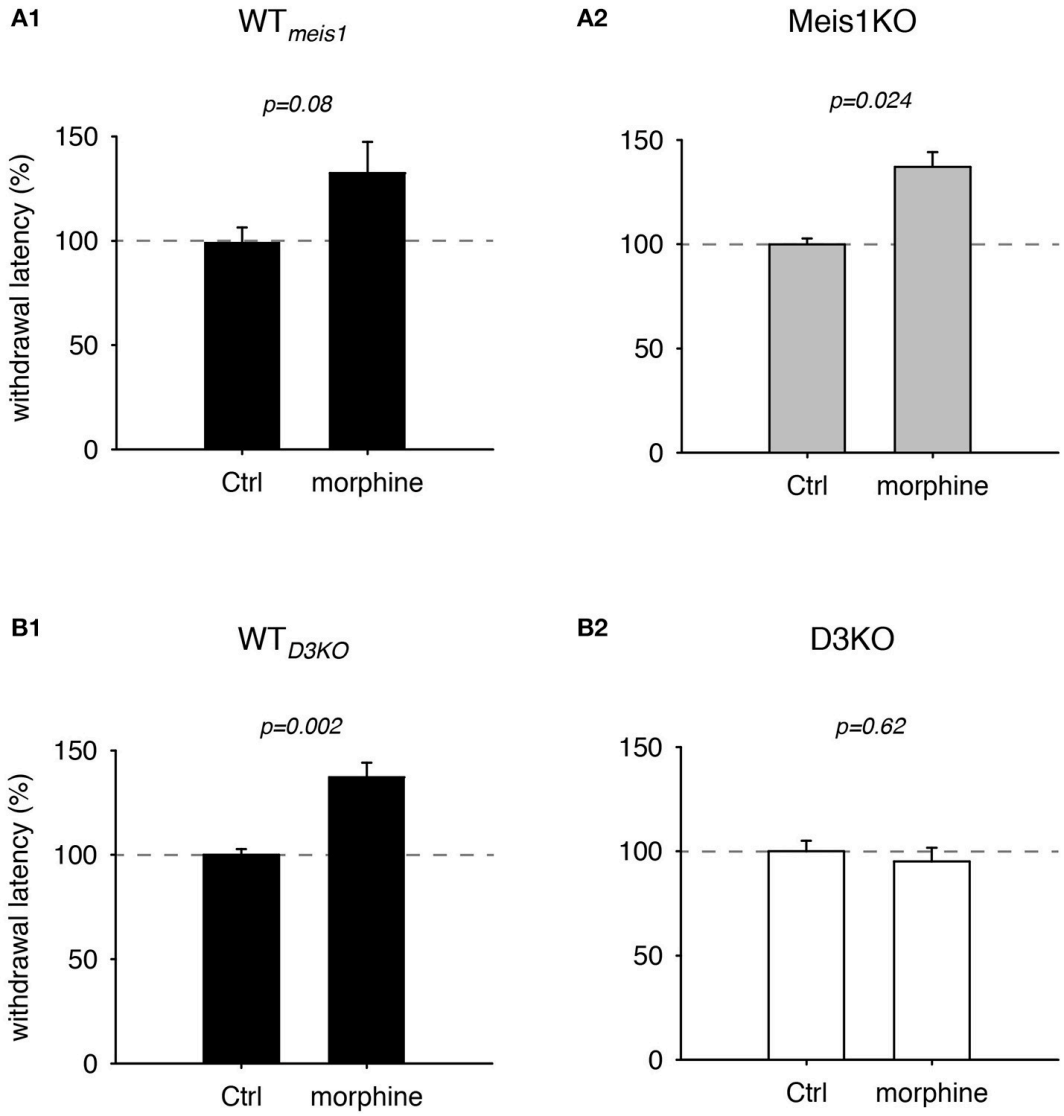
Thermal pain withdrawal latencies of WT, Meis1KO, and D3KO under sham (Ctrl) and morphine conditions. **(A1)** Normalized representation of data from WT_*meis*1_ animals. Morphine significantly increased withdrawal latencies. **(A2)** Normalized representation of data from the pooled Meis1KO animals. As in WT_*meis*1_, morphine significantly increased withdrawal latencies. **(B1)** Normalized representation of data from WT_*D*3*KO*_ animals. Morphine significantly increased withdrawal latencies. **(B2)** Normalized representation of data from D3KO animals. Unlike in both WT lines and Meis1KO, morphine had no effect on withdrawal latencies in D3KO.

While opioids are commonly used for treating chronic neuropathic pain, they have also become a treatment of choice in dopamine agonist-refractory RLS (Trenkwalder et al., 2015; Gemignani et al., 2016). As we have previously shown that the D3KO mouse expresses a morphine-tolerant phenotype if treated with low doses of morphine (Brewer et al., 2014), we next addressed the question if the Meis1KO mouse follows the WT or the D3KO phenotype when challenged with morphine (Figure 2). In Meis1KO cohort 1, WT withdrawal latencies increased from 6.8 ± 0.45 s to 9.1 ± 1.2 s (*n* = 6), while responses of Meis1KO increased from 6.3 ± 1.2 s to 10.1 ± 1.1 s (*n* = 7). Similarly, in cohort 2, WT withdrawal latencies increased from 6.5 ± 1.2 s to 9.3 ± 1.9 s (*n* = 5), while responses of Meis1KO increased from 6.3 ± 0.6 s to 7.9 ± 1.9 s (*n* = 7). After normalization to each WT_*meis*1_ control, the data are as follows: WT_*meis*1_; Ctrl: 99.3 ± 7.1%; after treatment: 132.5 ± 14.8%, *p* = 0.08, paired *t*-test, *n* = 11, power: 0.83, Figure 2A1; Meis1KO; Ctrl: 99.2 ± 5.7%; after treatment: 143 ± 16.6%, *p* = 0.024, paired *t*-test, *n* = 14, power: 0.59, Figure 2A2. For WT_*D*3*KO*_ the raw data were as follows; Ctrl: 8.3 ± 0.3 s; after treatment: 11.4 ± 0.6 s (*n* = 7), while D3KO were not responsive to 2 mg/kg morphine (Ctrl: 6.6 ± 0.4 s; after treatment: 6.1 ± 0.4 s, *n* = 7). After normalization to the pre-treatment control, the data for WT_*D*3*KO*_ are: 99.8 ± 2.8%; after treatment: 137.1 ± 7%, *p* = 0.002, paired *t*-test, power: 0.99; Figure 2B1. In contrast, normalized the data for the D3KO are: Ctrl 100.6 ± 5%; after treatment: 95.1 ± 6.6%, *p* = 0.62, paired *t*-test, power: 0.05, Figure 2B2). Together, these data suggest that WT_*meis*1_, WT_*D*3*KO*_, and Meis1KO animals respond similar to morphine, while D3KO are not affected.

### Dopaminergic modulation of withdrawal latencies

Dopaminergics are the first line of therapy in the treatment of RLS symptoms, and they can cover a range from L-DOPA to highly specific D3 receptor agonists. We therefore sought to first test the effects of L-DOPA on thermal withdrawal latencies in the different animal strains before testing the effects of the more selective receptor agonists and antagonists. We found that treatment with 10 mg/kg L-DOPA did not significantly alter withdrawal latencies in WT controls, Meis1KO or D3KO (data not shown).

**Figure 3:**
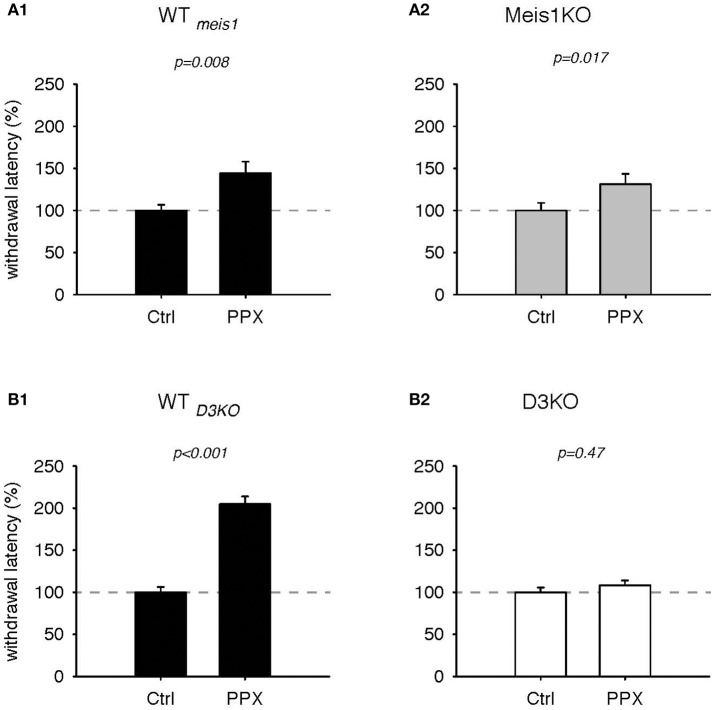
Thermal pain withdrawal latencies of WT, Meis1KO, and D3KO before and after D3 receptor agonist (PPX) treatment. **(A1)** Normalized representation of data from WT_*meis*1_ animals. PPX significantly increased withdrawal latencies. **(A2)** Normalized representation of data from the Meis1KO animals. As in WT_*meis*1_, PPX significantly increased withdrawal latencies. **(B1)** Normalized representation of data from WT_*D*3*KO*_ animals. PPX significantly increased withdrawal latencies. **(B2)** Normalized representation of data from D3KO animals. Unlike in both WT lines and Meis1KO, the D3 receptoer agonist PPX had no effect on withdrawal latencies in D3KO.

**Figure 4:**
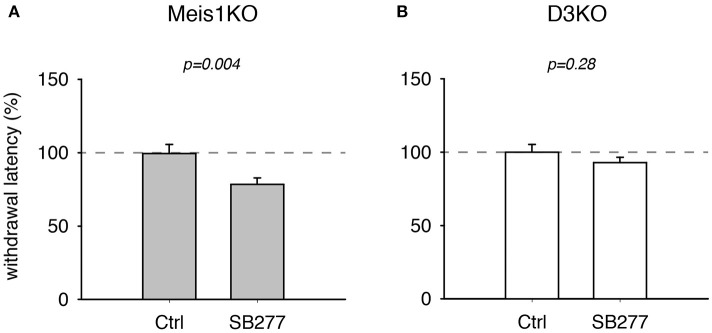
Thermal pain withdrawal latencies of Meis1KO and D3KO before and after treatment with the D3R antagonist SB 277011. **(A)** In Meis1KO, SB277 significantly decreased withdrawal latencies. **(B)** In D3KO, treatment with SB277 did not lead to any significant changes in withdrawal latencies.

We next tested the effects of the D3-receptor preferring agonist, pramipexole (PPX, Figures 3, 4). In WT_*meis*1_, treatment with PPX (0.5 mg/kg) increased withdrawal latencies from 100.2 ± 6.6% to 144.4 ± 13.7%, (*p* = 0.008, paired *t*-test, *n* = 11, power: 0.81, Figure 3A1; raw data for WT_*meis*1_ cohort 1: Ctrl 6.1 ± 0.7 s, PPX 9.6 ± 1.3 s, *n* = 6; cohort 2: Ctrl 7.2 ± 0.5 s; PPX: 8.6 ± 0.9 s, *n* = 5). In Meis1KO, the PPX treatment also led to a significant increase in withdrawal latencies from 100.1 ± 8.9% to 131.3 ± 12.3% (*p* = 0.017, paired *t*-test, *n* = 14, power: 0.65, Figure 3A2; raw data for Meis1KO cohort 1: Ctrl 6.7 ± 0.9 s, PPX 9.1 ± 1.2 s, *n* = 7; cohort 2: Ctrl 7.2 ± 0.9 s; PPX: 7.8 ± 1 s, *n* = 7). The effect of PPX was similar in the WT of the D3KO mice (WT_*D*3*KO*_) to the effect observed in WT_*meis*1_ and Meis1KO, but it did not alter the responses in the D3KO. In WT_*D*3*KO*_, withdrawal latencies rose significantly from 100 ± 6.3% (Ctrl) to 204.8 ± 9% (PPX, *p* < 0.001, paired *t*-test, *n* = 8, power: 1.0, Figure 3B1). In contrast, PPX did not have any significant effect in D3KO (Ctrl: 100 ± 5.6%, PPX: 108.4 ± 5.6%, *p* = 0.47, *t*-test, *n* = 6, power: 0.5, Figure 3B2). The raw data were as follows: WT_*D*3*KO*_; Ctrl 6.6 ± 0.5 s, PPX 12.9 ± 1.8 s, *n* = 8; D3KO; Ctrl 9.3 ± 0.6 s; PPX: 10.1 ± 0.8 s, *n* = 9.

Additionally, treatment with the D3 receptor antagonist, SB 277011, significantly decreased withdrawal latencies in Meis1KO but not D3KO (Figure 4). In Meis1KO, withdrawal latencies dropped from 99.5 ± 6.3% (Ctrl) to 78.5 ± 4.4% (SB277, *p* = 0.004, *n* = 7, paired *t*-test, power: 0.96, Figure 4A). However, we did not observe any significant effect in D3KO mice (Ctrl: 99.9 ± 5.3%, SB277: 92.8 ± 3.6%, *p* = 0.28, *n* = 6, paired *t*-test, power: 0.09, Figure 4B). The raw data were as follows: Meis1KO; Ctrl 7.2 ± 0.6 s, SB277 5.2 ± 0.2 s, *n* = 7; D3KO; Ctrl 7.9 ± 0.3 s; SB277: 7.8 ± 0.2 s, *n* = 6. Overall, treatment with the D3 receptor modulators led to similar effects in WT and Meis1KO and contrasted those in D3KO.

**Figure 5:**
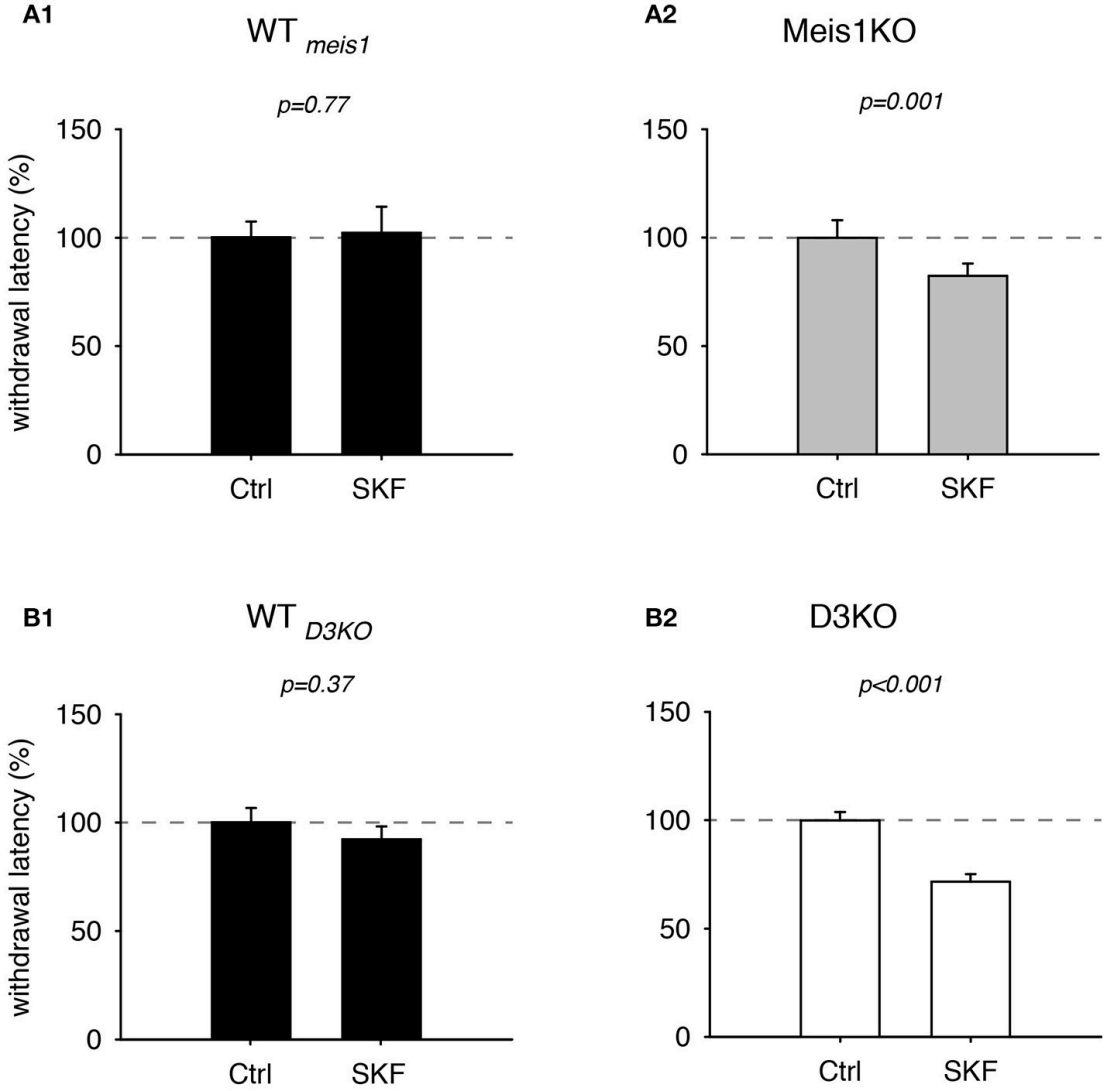
Thermal pain withdrawal latencies of WT, Meis1KO, and D3KO before and after D1 receptor agonist (SKF) treatment. **(A1)** Normalized representation of data from WT_*meis*1_ animals. SKF did not have a significant effect on withdrawal latencies. **(A2)** Normalized representation of data from the Meis1KO animals. SKF significantly increased withdrawal latencies. **(B1)** Normalized representation of data from WT_*D*3*KO*_ animals. SKF did not significantly alter withdrawal latencies in WT_*D*3*KO*_. **(B2)**. Normalized representation of data from D3KO animals. As in Meis1KO, the D1 receptor agonist significantly decreased withdrawal latencies in D3KO.

As recent findings from our lab point to a possible involvement of the D1 receptor in the face of D3 dysfunction (Brewer et al., 2014) or prolonged D3R agonist exposure (Dinkins et al., 2017), we next tested the effects of the D1 receptor agonist, SKF 38393 (SKF, Figure 5). We found that treatment with SKF had no significant effect in WT_*meis*1_ animals (Ctrl: 99.9 ± 8.2%; SKF: 92 ± 6.4%, *p* = 0.77, paired *t*-test, *n* = 5, power: 0.05, Figure 5A1), while the same treatment led to significant decrease of withdrawal latencies in Meis1KO (Ctrl: 99.9 ± 8.2%; SKF: 82.4 ± 5.7%, *p* = 0.001, paired *t*-test, *N* = 7, power: 0.98, Figure 5A2). The raw data were as follows: WT_*Meis*1_; Ctrl 6.6 ± 0.3 s, SKF 6.2 ± 0.5 s; Meis1KO; Ctrl 6.9 ± 0.5 s; SKF: 5.4 ± 0.3 s.

In WT_*D*3*KO*_ animals, SKF effects were similar to WT_*meis*1_ (Ctrl: 100.1 ± 6.6%, SKF: 92.2 ± 5.9%, *p* = 0.37, *n* = 5, power: 0.051, Figure 5B1), while the responses in D3KO were similar to those in Meis1KO (Ctrl: 99.8 ± 3.9%, SKF: 71.6 ± 3.6%, *p* < 0.001, *n* = 6, power: 1.0, Figure 5B2). The raw data were as follows: WT_*D*3*KO*_; Ctrl 16.8 ± 1.9 s, SKF 14 ± 1.2 s; D3KO; Ctrl 8.4 ± 0.9 s; SKF: 5.9±0.8 s. In contrast to the D1 receptor agonist SKF 38393, the D1 receptor antagonist, SCH 39166, displayed no differential effects in WT controls, Meis1KO or D3KO animals (WT_*Meis*1_ animals: Ctrl: 100.3 ± 7.2%, SCH: 102.3 ± 12%, *p* = 0.89, paired *t*-test, *n* = 5, power: 0.05, Meis1KO: Ctrl: 100.3 ± 6.9%, SCH: 110.6 ± 11%, *p* = 0.4, paired *t*-test, *n* = 7, power: 0.05; D3KO: Ctrl: 99.7 ± 5.5%, SCH: 111.6 ± 8.8%, *p* = 0.3, paired *t*-test, *n* = 6, power: 0.07). Taken together, the results of these dopaminergic modulators suggest that D3 receptor-mediated actions affect WT and Meis1KO similarly, while activation of D1 receptor signaling pathways exert similar effects in Meis1KO and D3KO but not WTs.

### D1R and D3R protein expression in the lumbar spinal cord of WT, Meis1KO, and D3KO animals

**Figure 6:**
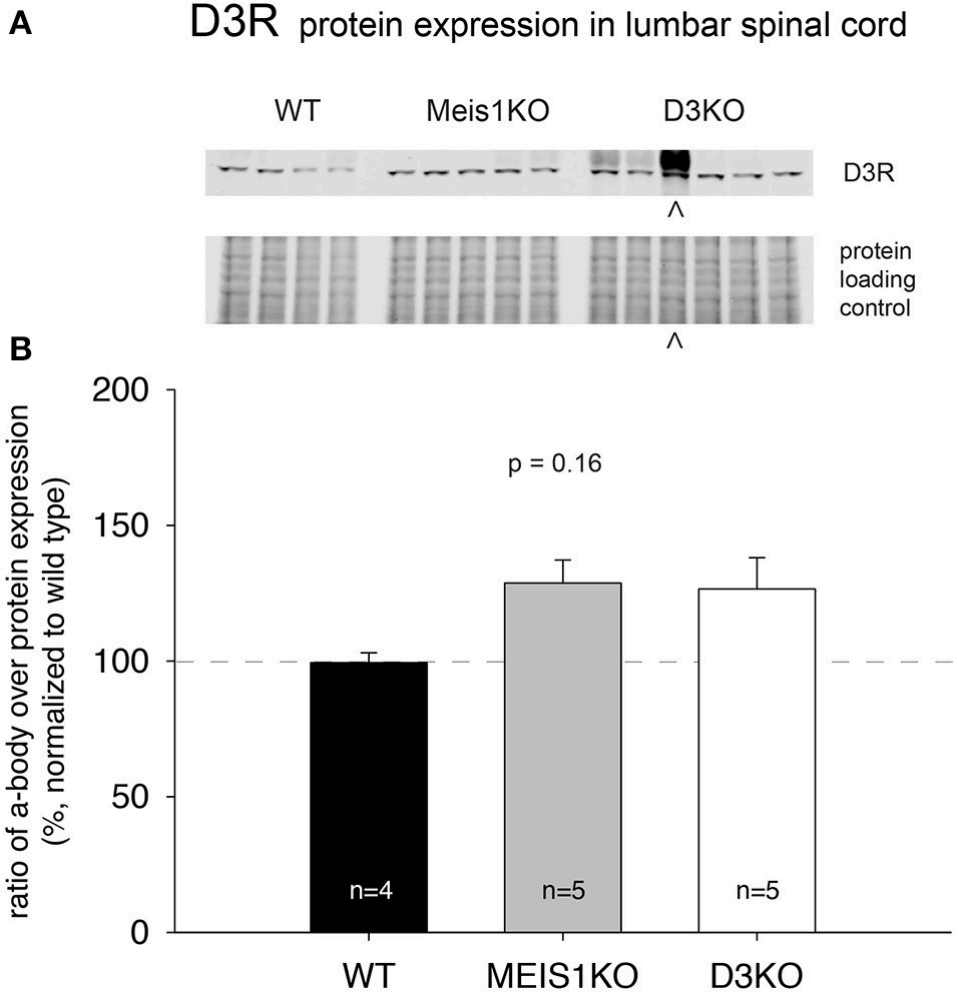
D3R expression in the lumbar spinal cord. **(A)**. Western blot of D3R protein expression in the spinal cord. Top: D3R protein band; bottom: protein loading control. **(B)**. Quantification of D3R protein expression in the spinal cord, normalized to protein loading control / lane. D3R expression was not significantly altered in Meis1KO and D3KO animals. ∧: D3KO lanes that were excluded from the subsequent analysis.

**Figure 7:**
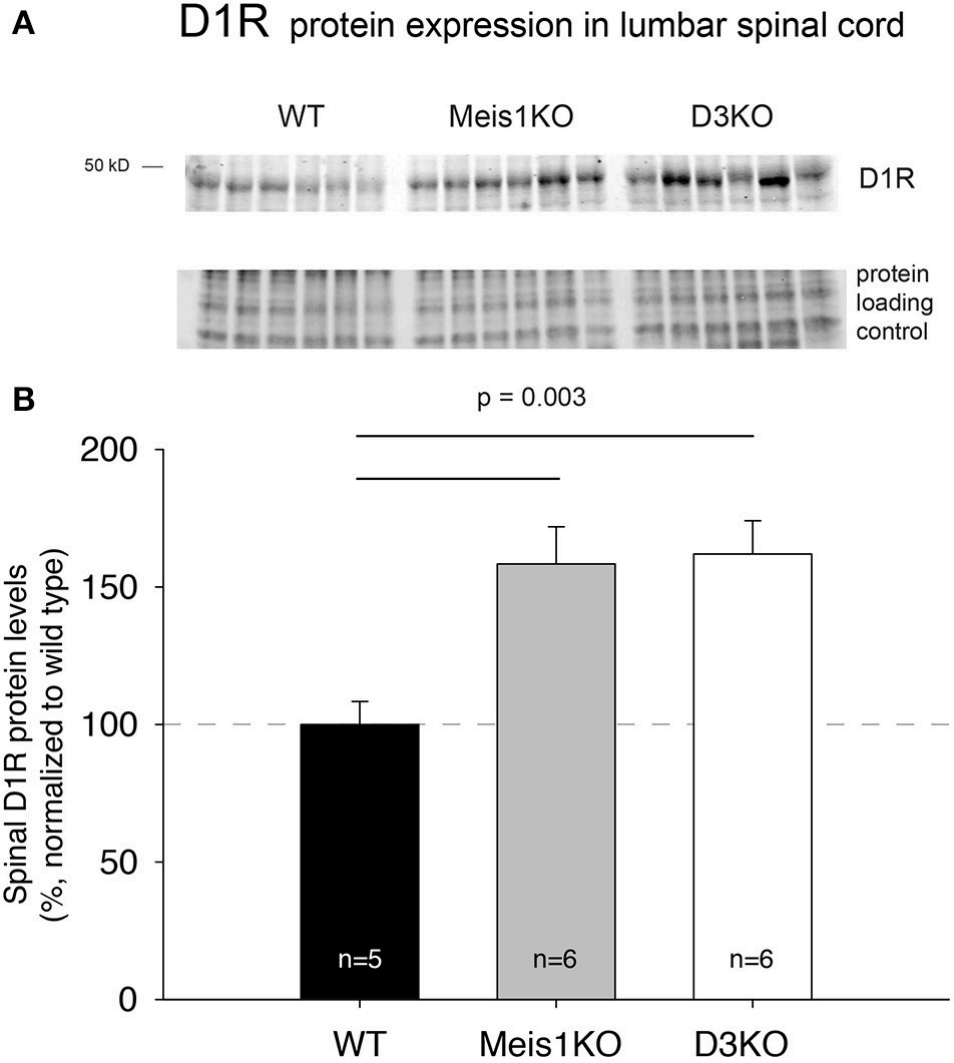
D1R expression in the lumbar spinal cord. **(A)** Western blot of D1R protein expression in the spinal cord. Top: D1R protein band; bottom: protein loading control. **(B)** Quantification of D1R protein expression in the spinal cord, normalized to protein loading control / lane. D1R expression was significantly increased in Meis1KO and D3KO over WT animals.

As treatments with D3- and D1 receptor-preferring agonists and antagonists respectively led to different outcomes, we next tested if the expression of these dopamine receptor subtypes was differentially regulated in Meis1KO and D3KO over WT (Figures 6, 7). We found that D3 receptor protein expression did not significantly differ between WT_*Meis*1_, Meis1KO, and D3KO (WT: 99.1 ± 3.5%; Meis1KO: 128.7 ± 8.5%; D3KO: 126.5 ± 11.5%, *p* = 0.16, One-Way ANOVA; Figure 6). Note that the D3KO expresses a dysfunctional D3 receptor that is not embedded into the membrane (Clemens et al., 2005). In contrast to the D3 receptor, when we probed for D1 receptor expression in the spinal cord, we found significantly increased D1 receptor protein levels in both Meis1KO and D3KO over WT controls (WT: 99.9 ± 8.4%; Meis1KO: 158.4 ± 13.5%; D3KO: 162 ± 12%, *p* = 0.003, One-Way ANOVA, Figure 7).

### Increased locomotor activity in Meis1KO and D3KO animals

**Figure 8:**
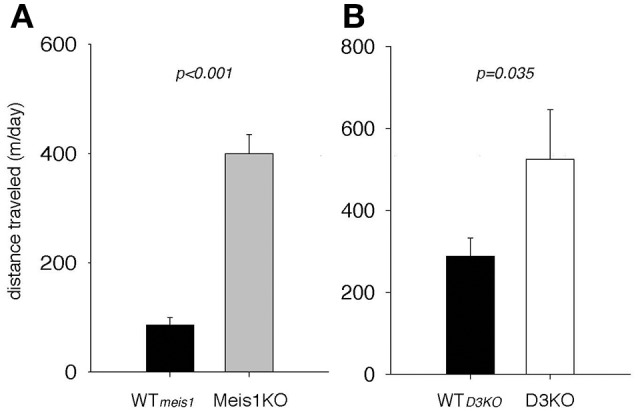
Spontaneous locomotor activities of Meis1KO and D3KO and their respective controls. **(A)** Meis1KO. Meis1KO displayed significantly increased locomotor activities per 24 h over their WT controls (WT_*Meis*1_). **(B)** Similar to Meis1KO, D3KO animals ran significantly longer distances per day over their controls (WT_*D*3*KO*_).

As the pharmacological and Western blot data suggested a role of the D1 receptor system in the control of spinal cord function, and as D1 receptor activation can recruit spinal cord networks to generate locomotor-like activities, we next tested if Meis1KO and D3KO show altered locomotor activities over their respective WT controls (Figure 8). We found that Meis1KO exhibited significantly increased locomotor activities compared to their WT (WT_*meis*1_: 86.1 ± 13.7 m/day, *n* = 7; Meis1KO: 399.7 ± 35.5 m/day, *n* = 8, *p* < 0.001, *t*-test, power: 1.0), and that D3KO showed a similar significant increase over their WT controls (WT_*D*3*KO*_: 343.2. ± 41.3 m/day, *n* = 16; D3KO: 587 ± 120 m/day, *n* = 8, *p* = 0.035, *t*-test, power: 0.48).

## Discussion

Here we compared thermal pain withdrawal latencies in two animal models of RLS, D3KO, and Meis1KO mice against wild type (WT) controls under different drug treatment conditions. We found that, under baseline conditions (sham), withdrawal latencies of WT and Meis1KO were similar, while those of D3KO were decreased, suggesting heightened excitability in these animals to a thermal stimulus and confirming earlier *in vivo* and *in vitro* studies (Keeler et al., 2012). DA acts via both excitatory (D1 and D5 receptor) and inhibitory (D2, D3, and D4 receptor) receptor subtypes, and D3KO animals express increased locomotor and rearing activities, indicating an inhibitory role of the D3 receptor in the control of these motor behaviors (Accili et al., 1996). Moreover, while present in all laminae of the spinal cord (Zhu et al., 2007), the D3 receptor is most densely expressed in the dorsal horn (Levant, 1998), suggesting a strong modulatory effect of D3 receptor-mediated pathways in this sensory area of the cord. Further, the lack of function of the inhibitory D3 receptor in the isolated spinal cord *in vitro* was sufficient to increase the number or large-reflex spinal reflex amplitudes when compared to WT controls (Clemens and Hochman, 2004). Similar to D3KO, Meis1KO show a pattern of hyperactivity but contrary to D3KO, express no significant nociceptive differences in a hotplate test (Salminen et al., 2017). This is consistent with our findings under baseline conditions where Meis1KO and WT express similar withdrawal thermal pain latencies, whereas reflex latencies are decreased in D3KO (Figure 1).

### Effects of morphine

Opioids are powerful modulators of nociceptive pathways, and we have shown previously that low morphine exposure (at 2 mg/kg, i.p.) was unsuccessful in modulating thermal pain withdrawal reflexes in D3KO and that this effect could be mimicked by acutely blocking D3 receptors in the isolated WT spinal cord preparation (Brewer et al., 2014). There, we also reported that the lack of a morphine effect in the D3KO animal was associated with an increased expression of the D1 receptor subtype in the spinal cord. While we did not perform other behavioral tests on pain (i.e. von Frey testing for mechanical pain), we confirmed the earlier D3KO findings here. We also found that Meis1KO and WT respond to morphine similarly (Figure 2). These data suggest that their opioid receptor pathways are alike, and preliminary Western blots indicate that the protein expression of the phosphorylated MOR is similar between WT and Meis1KO, but significantly increased in D3KO (data not shown). We are currently assessing if those receptor changes are found systemwide in the CNS or whether they are confined to specific (dorsal or ventral) areas of the spinal cord.

### Effects of L-Dopa

We used L-DOPA (levodopa) as the immediate precursor of DA, to test the effects of raised DA levels on sensorimotor function in the three animal lines but observed no clear effect in either animal line. While L-DOPA plays an important modulatory role in controlling, at least temporarily, RLS symptoms (Akpinar, 1982; Paulus and Schomburg, 2006; Stiasny-Kolster et al., 2013), it can exert differential effects on monosynaptic and oligo- or polysynaptic nociceptive and non-nociceptive reflexes, where it depresses monosynaptic reflexes of flexors but not extensors, but also depresses transmission in nociceptive flexor reflex pathways (Schomburg and Steffens, 1998). There are several not mutually exclusive scenarios that may explain lack of any significant effect in our experiments: i) we only tested L-DOPA at a single dose of 10 mg/kg thus it is possible that lower or higher doses would have yielded different results. However, doses of less than 10 mg/kg have been sufficient to provide behavioral effects and induce L-DOPA-induced dyskinesia in 6-hydroxydopamine lesioned mice (Lundblad et al., 2004). ii) Increasing dopamine levels with L-DOPA does not necessarily target the different DA receptor subtypes similarly. It is conceivable that high-affinity D3 receptor-activated inhibitory pathways compete with low-affinity D1 receptor-activated excitatory pathways, resulting in a net zero effect of this drug. iii), L-DOPA may act on the different components of sensory pain pathways differently; for example, in high spinal cats the onset of reflex facilitation induced by noxious radiant heat and mediated by A∂ fibers is delayed after injection of L-DOPA, while the late component persisted (Schomburg et al., 2011).

### Effects of D3R and D1R agonists and antagonists

While the conversion of L-DOPA to DA can lead to an activation of both inhibitory and excitatory receptor subtypes, we hypothesize that our findings reflect a strong influence of the D3 receptor in mediating the behavioral responses tested. The D3 receptor has a very high affinity to DA (Cote and Kuzhikandathil, 2014), thus an initial DA increase will primarily (but not exclusively) mediate an inhibitory response that should be missing in the D3KO. Both D3 and D1 receptors are expressed in the lumbar spinal cord (Zhu et al., 2007), and DA can up- or downregulate cellular and network functions in a dose-dependent manner (Missale et al., 1998; Thirumalai and Cline, 2008; Clemens et al., 2012). Current DA-based RLS treatment options center around D3 receptor agonists (Ferini-Strambi et al., 2016; Ferré et al., 2017), but their effect is reduced over time and can cause a worsening of the symptoms (augmentation) (Allen et al., 2011; García-Borreguero and Williams, 2011; Earley et al., 2017; Trenkwalder et al., 2017). While we have previously shown that D3 receptor agonists and antagonists can oppositely regulate spinal reflex amplitudes (SRAs) of WT *in vitro* while they do not alter SRAs in D3KO (Clemens and Hochman, 2004), we here wanted to test how these neuromodulators act *in vivo*, and how their effects compare with the Meis1KO animals (Figure 4). Treatment with both D3 receptor modulators (agonist pramipexole; PPX, and antagonist SB277011-A; SB277) led to nearly identical outcomes in WT and Meis1KO respectively, and had, as expected, no effect in the D3KO. In WT and Meis1KO, PPX had a strong analgesic effect that was counteracted by SB277, suggesting a similar role for the D3 receptor in modulating the thermal nociceptive pathway in these two animal strains. As the D3 receptor in the spinal cord is most prominently expressed in the sensory neurons of the dorsal horn (Levant, 1998), it is tempting to speculate that reduced DA levels at this sensory interface in the evening or at night (Carlsson et al., 1980; Sowers and Vlachakis, 1984) might reduce descending inhibitory control and thus contribute to the emergence of the circadian symptoms in RLS (“urge to move”). Of particular note is that such a reduced functional state of the D3 receptor in the dorsal horn could also arise independent of circadian DA levels, either by Meis1-dependent compromised projections of descending DA fibers from the A11 nucleus (M. Aschner, personal communication) or with the normal aging-related gradual decline of DA levels (Haycock et al., 2003) and a subsequent reduction in the expression levels of inhibitory G*i*-coupled DA receptors (Mesco et al., 1991; Valerio et al., 1994). As with the circadian DA fluctuations, low DA levels would first affect the D3R and thus reduce overall DA-mediated inhibition. The D3KO animal then could serve as a model to specifically assess the mechanisms that follow a D3 receptor dysfunction in the sensory part of the spinal cord.

In contrast to D3 receptor modulators, the effects of the D1 receptor agonist was similar between Meis1KO and D3KO, but had no effect in WT. We suspect that the increase in spinal D1R protein expression (discussed below) may be the component that drives this behavioral outcome. We here confirmed that both Meis1KO and D3KO animals express increased locomotor activities (Accili et al., 1996; Salminen et al., 2017), and there is ample evidence that, in the isolated spinal cord preparation, D1R agonists can activate the central pattern generator (CPG) for locomotion (Kiehn and Kjaerulff, 1996) (Starr and Starr, 1993; Lapointe and Guertin, 2008; Urs et al., 2011; Sharples et al., 2015). As the locomotor CPG is contained to the ventral aspect of the thoraco-lumbar spinal cord (Kjaerulff and Kiehn, 1996; Kiehn, 2006, 2016) and the D1 receptor is more strongly expressed in ventral than sensory areas of the spinal cord (Zhu et al., 2007), D1 receptor-mediated actions may predominantly target motor-related over sensory functions in the spinal cord. Intriguingly, periodic limb movements during sleep (PLMS) are often associated with RLS (Wetter and Pollmächer, 1997; Moore et al., 2014; Li et al., 2016), and PLMS scores are regularly used to quantify RLS severity (Happe et al., 2001; Manconi et al., 2011b; Winkelmann et al., 2017). The similar responsiveness of Meis1KO and the D3KO to the D1 receptor modulators and their heightened motor activity suggest that the excitability of the spinal CPG may be upregulated in these animals, possibly via a D1 receptor-dependent beta-arrestin 2/phospho-ERK signaling complex that selectively mediates the locomotor CPG (Urs et al., 2011), which then could provide a target to understand the development of PLMS in future mechanistic studies.

### D3R and D1R protein expression in the spinal cord

All DA receptor-mRNAs are expressed in the neurons of the rodent spinal cord (Zhu et al., 2007), although it is unclear if this translates also to proteins similarly, and if it is also the case in primates and man. For example, in non-human primates the D1 receptor is missing in the cord (Barraud et al., 2010), however its functions may be compensated for by the D5 receptor subtype, which also activates adenylyl cyclase (Missale et al., 1998). Using standard Western blot techniques, we tested for D3 receptor and D1 receptor protein expression in the lumbar spinal cord of the three animal models tested behaviorally. While we found no significant difference in D3 receptor expression across WT, Meis1KO and D3KO (Figure 6), we observed a significant upregulation of D1 receptor protein expression in Meis1KO and D3KO over WT (Figure 7). While present at relatively low levels in the spinal cord (Zhu et al., 2007), quantitative autoradiography has revealed that the D3 receptor is predominantly expressed in the dorsal horn (Levant, 1998), where it is in a prime position to modulate sensory pathways. In contrast, D1 receptor activation is regularly used to induce fictive locomotion in the isolated spinal cord preparation (Lapointe and Guertin, 2008; Han and Whelan, 2009; Sharples et al., 2015), suggesting a strong influence of the D1 receptor system to activate these more ventrally located circuits. Thus an increase in the availability or activation of D1 receptors in D3KO animals could explain an increase in an overall increase in the overall excitability and activity, similar to that observed with normal aging (Keeler et al., 2016).

The presence of a D3 receptor protein in the D3KO may appear unexpected, but the D3KO mouse used in our studies is not a genetic knockout of the D3 receptor; rather it is a targeted mutation of the D3 receptor gene in the second intracellular loop of the predicted protein sequence that prevents the incorporation of the D3R into the membrane (Accili et al., 1996). While this mutation does not preclude the transcription and translation of the D3 receptor, it prevents its insertion into the cell membrane (Zhu et al., 2008), rendering it functionally inactive (Clemens and Hochman, 2004). The lack of a difference between WT and Meis1KO supports the behavioral effects of the D3 receptor compounds and underlines the similarities between these two models with regard to the modulation of their sensory circuits.

In contrast to the D3 receptor data, the results of the D1 receptor protein expression point to a similarity of Meis1KO and D3KO, but not with either of them and the WT. We have shown previously that D3KO express not only decreased thermal pain withdrawal latencies but also an increased spinal D1 receptor protein expression (Brewer et al., 2014), and it is possible that in these animals a lack of synergistic D1-D3 receptor intra-membrane receptor-receptor interactions may account for the underlying mechanism that is responsible for this behavioral outcome (Marcellino et al., 2008). However, our data do not completely answer the question if there is a spatial component to the differences in D3 receptor and D1 receptor protein expression in the cord between the two genetically-modified animals and their WT controls. While we performed our Western blot experiments on lumbar spinal segments only, we did not anticipate the outcome that point to potential differential changes in sensory and motor circuits, and hence did not further dissect out and differentiate the outcomes between ventral versus dorsal aspects within those segments.

### The caveat of using dopaminergics *in vivo* to test sensorimotor circuits in the spinal cord

Descending DA projections to and the presence of DA receptors in the spinal cord are well established (Holstege et al., 1996; van Dijken et al., 1996; Levant, 1998; Zhu et al., 2007), yet we cannot exclude the possibility that any of the drugs tested may have triggered changes in descending pathways, which in turn altered spinal reflex circuit excitabilities. Possible alternate approaches to test whether the DA effects are systemwide or truly spinal, which was beyond the scope of this study, would be to test the modulatory effects of these compounds in acutely anesthetized and spinalized animals, or by employing intrathecal drug delivery or conditional knockout approaches. In fact, data from a preliminary study, in which a D3 receptor-specific blocker was applied intrathecally, indicate that such an intrathecal approach alone can alter locomotor patterns and spinal D1 receptor expression (Jensen et al., 2014).

### D3KO and Meis1KO–complementary models of RLS?

**Figure 9:**
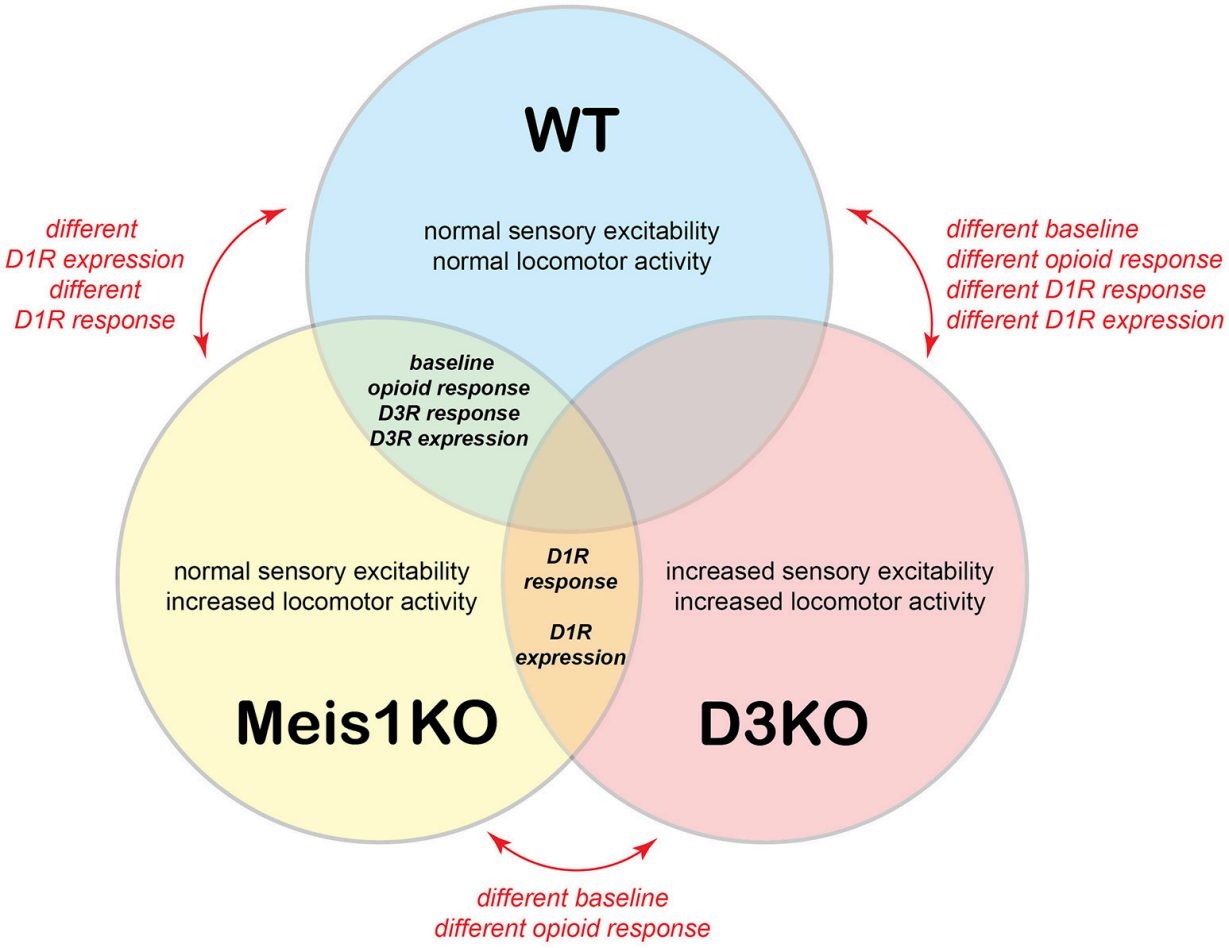
Model of relationships between WT, Meis1KO, and D3KO animals. WT and Meis1KO share a similar baseline under control conditions, respond similarly to opioids, and have a similar D3R expression in the spinal cord. However, they differ in their responses to D1R modulators. WT and D3KO have different baselines under control conditions and respond differently to opioids, D3R, and D1R modulators. Meis1KO and D3KO have different baselines under control conditions, but respond similarly to D1R modulators, and have similar D1R expression levels in the spinal cord.

Figure 9 presents a comparative model, in which we summarize baseline properties, behavioral responses to the drugs tested, and D1 and D3 receptor protein expression data between WT, Meis1KO, and D3KO. WT and Meis1KO are similar with regard to sensory excitability at baseline (sham), their behavioral responses to morphine and D3 receptor modulators, and the expression levels of the spinal D3 receptor. Importantly, WT are different from D3KO with regard to baseline, opioid response, D1 receptor modulators, and D1 receptor expression. While Meis1KO and D3KO differ from each other in baseline and opioid response, they react similarly to D1 receptor modulators, and they express similarly increased D1 receptor protein expression levels in the lumbar spinal cord. As Meis1KO show at baseline only an increased locomotor activity, but normal sensory excitability, and as locomotor circuits are located in the ventral horn of the spinal cord, we posit that the Meis1KO mouse may serve as a model to specifically explore the mechanisms affiliated with the motor-related aspects of RLS (i.e. PLMS). In contrast, the increased sensory excitability and locomotor activity in D3KO suggest that both sensory and motor circuits are functionally upregulated in the spinal cord of this mouse, and that this model may be used to examine the impact of both sensory and motor pathways affiliated with RLS.

## Author contributions

SM, M-LD, SL, YunL, C-TL, and MK performed the experiments. SM, M-LD, MK, C-TL, and SC analyzed the data. SC and YuqL designed the experiments. SM, KB, YuqL, and SC wrote the manuscript. SM, M-LD, MK, SL, YunL, YuqL, C-TL, KB, YL, and SC approved of the manuscript.

### Conflict of interest statement

The authors declare that the research was conducted in the absence of any commercial or financial relationships that could be construed as a potential conflict of interest.
